# Waves of prediction

**DOI:** 10.1371/journal.pbio.3000426

**Published:** 2019-10-03

**Authors:** Karl J. Friston

**Affiliations:** The Wellcome Centre for Human Neuroimaging, UCL Queen Square Institute of Neurology, London, United Kingdom

## Abstract

Predictive processing (e.g., predictive coding) is a predominant paradigm in cognitive neuroscience. This Primer considers the various levels of commitment neuroscientists have to the neuronal process theories that accompany the principles of predictive processing. Specifically, it reviews and contextualises a recent *PLOS Biology* study of alpha oscillations and travelling waves. We will see that alpha oscillations emerge naturally under the computational architectures implied by predictive coding-and may tell us something profound about recurrent message passing in brain hierarchies. Specifically, the bidirectional nature of forward and backward waves speaks to opportunities to understand attention and how it nuances bottom-up and top-down influences.

## Abstract 2

This Primer explores the electrophysiological concomitants of predictive processing in the brain, with a special focus on a recent study on alpha rhythms and travelling waves, and what they tell us about message passing in cortical hierarchies.

## Introduction

It is difficult to find a contemporary paper in cognitive neuroscience that does not defer to the notion of predictive processing and associated schemes like predictive coding that implement predictive processing in cortical and subcortical hierarchies. For people not familiar with the rhetoric in this field, it is useful to distinguish between the principles of predictive processing and the neuronal process theories that might implement them. There are 2 approaches to predictive processing: the low road usually starts from Kantian notions and Helmholtz’s formulation of perception as unconscious inference [1]. The basic idea is that the brain is a constructive organ, actively generating explanations for the sensorium and then testing its hypotheses against sensory data. This notion underwrites predictive coding in the brain [2,3], a scheme originally developed to compress sound files in the 1950s.

**Fig 1:**
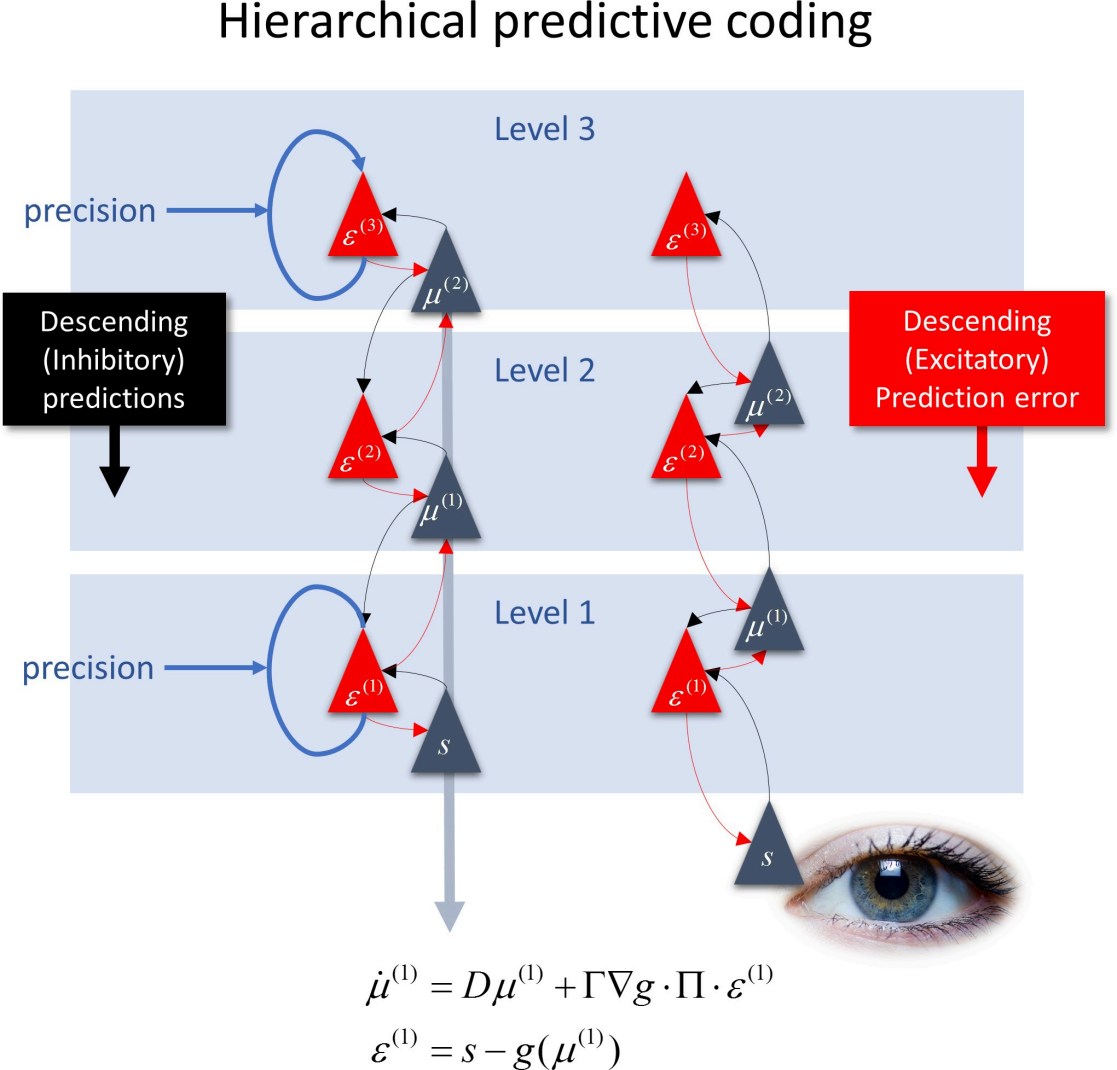
This figure illustrates the basic architecture of message passing in hierarchical predictive coding. Here, 3 hierarchical levels are shown in schematic form (blue boxes). These levels are populated by pairs of populations—encoding prediction errors (red triangles) and expectations (blue triangles). Sensory input enters at the lowest level of the hierarchy (denoted by the eye). The equations describe the mathematical form of predictive coding, expressed as a Kalman-Bucy filter. Here, μ represents an expectation (i.e., mean) of some state of the world. Conversely, *ε* represents prediction error, which is just the difference between sensory input *s* and predictions of that input under some generative model *g*, given expected states of affairs. This form means that prediction errors are weighted by their precision Π to drive expectations, which, in turn, supply predictions to error units, thereby suppressing them. The architecture on the left is the canonical or standard architecture [6], in which prediction errors ascend from one level to the next and are complemented by a descending counter-stream of predictions. However, exactly the same message passing can be implemented by simply moving the expectation units down a little (depicted by the blue arrow) to produce the architecture on the right. Although nothing changes in terms of inference, now the predictions ascend the hierarchy, while prediction errors are conveyed by extrinsic (between cortical level) connections. The self-connections (in blue) stand in for a precision or gain control that modulates the disinhibition of error units. The precision of prediction errors at different hierarchical levels can have profound effects on message passing and subsequent belief updating or evidence accumulation. Please see main text.

Predictive coding is appealing in its simplicity: essentially, it sets up a number of competing expectations about the causes of sensory input and then revises or updates these expectations on the basis of prediction errors. These errors are just the difference between what was predicted and what is actually observed. The ensuing belief updating can then be expressed as a recursive exchange of signals between neuronal populations encoding expected states of the world generating sensations and prediction errors. When predictions are generated under a hierarchical (generative) model of how (hidden) states of the world cause other states, we have a message-passing scheme that looks very much like the recurrent exchange of signals in visual cortical hierarchies; with ascending (prediction error) connections and a descending (prediction) counter-stream (Fig 1). Predictive processing has emerged as an enactive generalisation of this idea to encompass action (through the introduction of motor and autonomic reflexes) and applying the underlying (Bayesian) principles to planning and policy selection [4,5].

An alternative (high road) starts with the variational principles that underwrite self-organisation and assembly in sentient systems, to show that such systems can always be cast as making Bayesian inferences about their world, i.e., self-evidencing [5,7]. This leads to the notion of active inference, which can be regarded as a first principle account of predictive processing [8]. The key thing here is that active inference and predictive processing inherit from first principles, whereas schemes like predictive coding are particular (neuronal) process theories about how these principles are manifested in the brain. This means that all the heavy lifting—in terms of asking the right empirical questions—pertains to how predictive processing is implemented.

At present, predictive coding is the prime candidate for predictive processing, largely in virtue of its remarkable power in explaining extrinsic hierarchical connections and the intrinsic connectivity of cortical microcircuits. However, predictive coding is just a theory, and there are an increasing number of variants. Indeed, there are little ‘representation wars’ within the field. For example, are prediction errors or predictions passed forward from a lower level to a higher level [6,9–11] (see Fig 1)? Or do we need separate neuronal populations to encode positive and negative prediction errors [8,12]? These issues matter when it comes to finding definitive empirical evidence for the computational architectures entailed by predictive coding. So what would we expect to measure if predictive coding was the right kind of theory?

## Predictions of predictive coding

There are many aspects of functional architectures and neurophysiology that might underwrite predictive coding. We will focus on electrophysiological oscillations. What would predictive coding predict about oscillations in the brain? There are a few rules with which all variants of predictive coding must comply. These include the existence of separable neurons or neuronal populations encoding expected states of the world and prediction errors. Crucially, for every expectation unit there is an accompanying error unit. Furthermore, expectation units only provide afferents to error units and vice versa. Furthermore, these recurrent connections must possess the form of a negative feedback loop [13]. This holds irrespective of whether the reciprocal connections are intrinsic (within a cortical level) or extrinsic (linking different hierarchical levels) (see Fig 1).

With this picture in mind, one can conceive of distributed neuronal processing as being mediated by coupled oscillatory pairs of expectation and error units, such that error units excite expectation units and expectation units inhibit error units (polysynaptically). The implications for electrophysiology can be unpacked under 2 complementary characterisations of neuronal responses, namely, evoked or induced responses. Put simply, evoked responses are measured with the power of the average, while induced responses correspond to the average of the power. From the perspective of evoked responses (e.g., event related or local field potentials), one can imagine a sensory input perturbing a sequential or centrifugal hierarchy of coupled oscillators to produce a damped oscillatory response. The characteristic frequency of this response lies in the alpha range, with peaks separated by about a hundred milliseconds. Indeed, this characteristic response to stimuli has led to a whole industry, looking at successive peaks (e.g., N1, P2, etc.). From the current perspective, these evoked transients are exactly what would be predicted if recurrent message passing was in the game of resolving prediction errors—and suppressing the activity of error units (usually associated with superficial pyramidal cells) [6,10]. Clearly, the form of these transients will depend upon the hierarchical structure of message passing, such that (endogenous) components later in peristimulus time are consequent upon a penetration deep into the hierarchy and recurrent expression at lower (sensory) levels.

An alternative perspective on oscillatory dynamics is provided by induced responses, either characterised as a function of frequency over peristimulus time or in continuous recordings. In this arena, the predictions of predictive coding become more nuanced, with a focus on spectral asymmetries between forward and backward message passing [14–16]. The current story is that ascending (forward) prediction errors are conveyed at a faster frequency (e.g., gamma), while backward (descending) predictions are manifest at lower frequencies (e.g., alpha and beta).

The thing that predictive coding brings to the table here is a fundamental asymmetry in the construction of predictions and prediction errors. This follows because predictions are based upon interactions between the multifaceted causes of our sensorium [17]. For example, if I wanted to predict current visual input, I would have to combine expectations about what was generating my visual signals and where that object was. In short, I would have to use a nonlinear mixture of descending predictions afforded by expectations of what and where. Mechanistically, this interaction entails nonlinear transformations at the level of intrinsic connectivity and intralaminar synaptic exchanges. So why is this important for induced responses? Any nonlinearity of this sort converts a low frequency into a high frequency; one can see this easily by imagining what would happen if you squared a sine wave to double its frequency.

This means that the prediction errors, based upon nonlinear mixtures of expectations, must express higher frequencies than the expectations that generate predictions. This spectral asymmetry has now become a meme in many branches of neuroscience, ranging from the role of attention in visual neuroscience [15] to the mechanisms that undergird speech perception in the auditory cortex [16]. This basic asymmetry—and implicit cross frequency coupling—leads to interesting questions about the underlying synaptic physiology and neurotransmitter basis of predictive coding. An important theme here is the role of neuromodulation in augmenting or attenuating the excitability of superficial pyramidal cells, which may broadcast prediction errors [6,10]. This theme of selective neuromodulation—at different levels of hierarchical message passing—will become particularly prescient later, when considering the relative influence of top-down and bottom-up afferents. So far, we have only considered the electrophysiological correlates of predictive coding in the time domain. But what about space and functional anatomy?

## Waves of prediction

**Fig 2:**
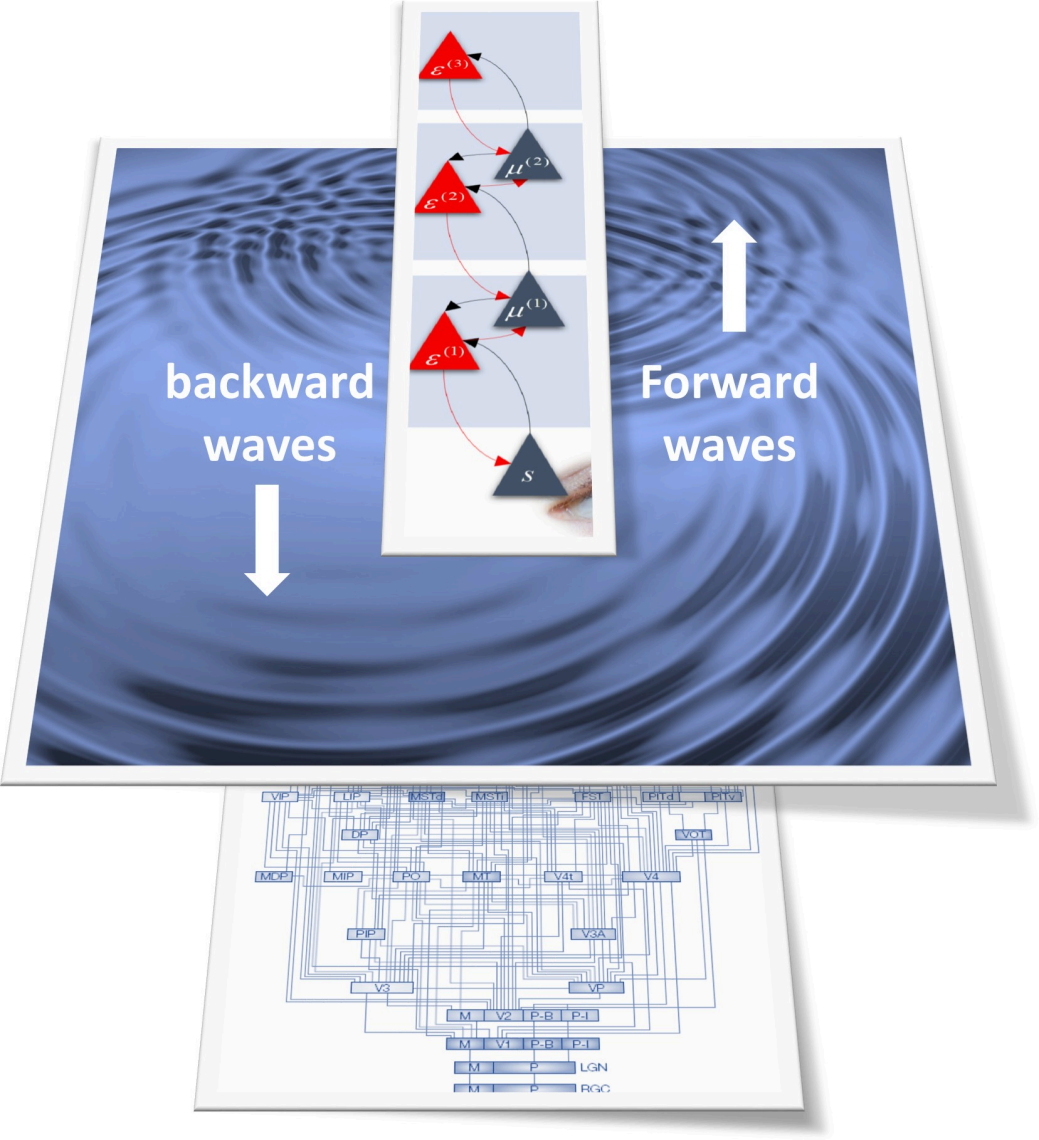
This schematic illustrates forward and backwards travelling alpha waves as they are envisaged to transverse the cortical hierarchy. The schematic on the top borrows from Fig 1 and illustrates a succession of reciprocally coupled populations encoding errors (red triangles) and expectation units (blue triangles). Following a sensory perturbation, prediction errors ascend the hierarchy, producing a wave of excitation that is ‘reflected’ at each level to produce wavelike spatiotemporal dynamics. This is illustrated here in terms of waves in water that could stand in for the ‘active matter’ that constitutes coupled neuronal oscillators, when densely packed.

At this point, the results in Alamia and VanRullen [18] acquire a particular importance, as we will now see. What does predictive coding have to say about the spatiotemporal propagation of dynamics over cortical hierarchies? One way to answer to this question is to build a minimal model of predictive message passing and ‘ping’ it with a stimulus to simulate a spatiotemporal response. This is precisely what the authors did—and then looked for evidence of alpha waves travelling up and down the hierarchy. Intuitively, one can imagine dropping a stone into a pond and eliciting concentric (alpha) waves spreading from the point of perturbation (Fig 2). However, we also have to factor in recurrent message passing, as waves are reflected from (hierarchically deployed) ‘edges’ of the pond back to the epicentre.

If we associate a sensory stimulus with a local perturbation at the ‘lower’ end of the pond, one might expect to see a predominance of ‘forward’ travelling waves. Conversely, if we perturb the ‘upper’ end, the waves would appear to travel in a ‘backwards’ direction. However, in both cases, waves will travel in both directions, due to recurrent connections. This is exactly what the authors established, using numerical analyses (and analytic solutions to differential equations that embody a simplified version of predictive coding) [18]. In short, hierarchical predictive coding suggests that forward and backwards travelling waves should be seen in response to sensory stimulation and top-down perturbations, respectively. Not only were the authors able to demonstrate that these dynamics could be recovered with a careful (Fourier) analysis of synthetic responses, they went on to show that the same phenomenology could be recovered from empirical electroencephalographic (EEG) signals [18]. Clearly, there are many message-passing schemes that could account for travelling waves. However, the point is that this particular spatiotemporal pattern of evoked responses was predicted under minimal assumptions, afforded by the predictive coding hypothesis.

There are many details that attend this finding. For example, (in silico) travelling waves could only be elicited in the alpha range with a particular set of synaptic time constants (and axonal conduction delays) mediating the response of expectation units to error units. Crucially, the range of these parameters matched almost exactly with empirical observations, namely, extrinsic conduction delays of about 12 ms and a lumped synaptic time constant of about 20 ms. An interesting point here is that these characteristic (membrane) time constants are greater than one would expect when considering a spiking neuron (i.e., 5–20 ms.). As intimated in Alamia and VanRullen [18], this tells us something interesting about neuronal implementations of predictive coding. This follows from the fact that the effective time constants of population dynamics are typically greater than any constituent neuron. This suggests that predictive coding may implemented at the level of ensemble or population averages, which speaks to an interesting debate about the nature of population codes in a Bayesian setting [19]. An intriguing aspect of the results of Alamia and VanRullen [18] was the sensitivity of forward and backwards travelling waves to the (lower and upper) levels of perturbation. We will close by pursuing this, because of its special relevance for understanding the nature of hierarchal inference, its implications for higher cognitive functions, and the pathophysiology that may attend neuropsychiatric conditions [20,21].

## The importance of being precise

The evidence for forward over backward travelling waves depends upon whether bottom-up sensory perturbations are supplied or higher-level activity was manipulated (c.f., changes in prior expectations). This was emulated in silico by switching on and off bottom-up versus top-down perturbations. In in a physiological setting, the sensitivity of prediction error units to their afferents holds the key for this selective gating and implicates all the neuromodulatory mechanisms mentioned above. This is an important aspect of predictive coding that takes us into the world of selective attention and sensory attenuation [10,21,22].

In brief, a key determinant—of the balance between descending predictions and ascending prediction errors—rests upon the precision or confidence afforded prediction errors. In short, if a prediction error conveys precise, newsworthy information, it will be amplified so that it has a greater impact on belief updating in subsequent hierarchical levels. Conversely, in noisy (or dark) unreliable settings, sensory precision can be attenuated, thereby emphasising top-down prior expectations over impoverished sensory evidence. This is just an expression of Bayes optimal inference. From a physiological perspective, the excitability of prediction error units—and their neuromodulatory control—takes centre stage in this selective gating. Finally, from a psychological perspective, we have an appealing metaphor for attentional selection and sensory attenuation. So why is this important for the spatiotemporal signatures of predictive coding?

As noted by Alamia and VanRullen [18], certain changes in attentional set will increase the precision or excitability of prediction error units lower in the hierarchy. In turn, this would increase the predominance of forward travelling waves. Conversely, in the absence of precise sensory information, or when holding unduly precise prior beliefs about the causes of our sensorium, one might anticipate a greater preponderance of backward travelling waves. This is clearly a prediction that could be addressed empirically, using the analyses described by Alamia and VanRullen [18] and manipulations of attentional set. This may be particularly exciting because it also affords the opportunity to quantify people's ability to switch attentional set or indeed engage sensory attenuation when appropriate. I focus on this opportunity, because many psychiatric syndromes have been associated with a failure of sensory attenuation or an inability to modulate the precision of various prediction errors [4,20,21].

## Abbreviation

EEG: electroencephalographic.
